# Physiological and Transcriptional Analysis Reveals the Response Mechanism of *Camellia vietnamensis* Huang to Drought Stress

**DOI:** 10.3390/ijms231911801

**Published:** 2022-10-05

**Authors:** Shuaishuai Shen, Wuping Yan, Shuao Xie, Jing Yu, Guanglong Yao, Pengguo Xia, Yougen Wu, Huageng Yang

**Affiliations:** 1 Hainan Yazhou Bay Seed Laboratory, Sanya Nanfan Research Institute of Hainan University, Sanya 572025, China; 2 Engineering Research Center for the Selection and Breeding of New Tropical Crop Varieties of Ministry of Education, College of Tropical Crops, Hainan University, Haikou 570228, China; 3 College of Horticulture, Hainan University, Haikou 570228, China; 4 School of Agricultural Sciences, Jiangxi Agricultural University, Nanchang 330045, China; 5 Key Laboratory of Plant Secondary Metabolism and Regulation of Zhejiang Province, College of Life Sciences and Medicine, Zhejiang Sci-Tech University, Hangzhou 310018, China

**Keywords:** *Camellia vietnamensis* Huang, drought stress, flavonoid biosynthesis, phenylpropanoid biosynthesis, phytohormone

## Abstract

Drought stress is considered the main obstacle restricting *Camellia vietnamensis* Huang (*C. vietnamensis*) yield. Hainan is the southernmost distribution region of *C. vietnamensis* in China and experiences a drought period annually. To study the drought-stress-response mechanism of *C. vietnamensis*, we treated seedlings of drought-tolerant (HD1) and drought-sensitive (WH1) cultivars with PEG-6000 (PEG) to simulate drought stress and compared the physiology and transcriptome of their leaves at 0 d, 3 d and 6 d posttreatment. Under drought stress, the growth of *C. vietnamensis* was inhibited, the relative water content (RWC) of leaves decreased and the contents of malondialdehyde (MDA), antioxidant enzyme activities, osmotic regulatory substances and secondary metabolites increased. Compared with those of WH1, the leaf RWC, osmotic-regulation substance content (proline, soluble protein and soluble sugar) and antioxidant enzyme activity (superoxide dismutase, peroxidase and catalase) of HD1 were significantly increased, while the relative electrical conductivity and MDA content were significantly decreased. Compared with WH1, 2812, 2070 and 919, differentially expressed genes (DEGs) were detected in HD1 0 d, 3 d and 6 d posttreatment, respectively, and the number of DEGs increased with increasing treatment time. The detected DEGs are involved in the drought stress response of *C. vietnamensis* mainly through plant-hormone signal transduction and lignin and flavonoid biosynthesis pathways. Drought stress significantly activated the expression of several lignin and flavonoid biosynthesis genes in HD1. Moreover, total flavonoid and total polyphenol contents in HD1 were significantly increased, suggesting that the accumulation of flavonoids may be a key factor in the drought stress response of *C. vietnamensis*. Additionally, 191 DEGs were associated with coding transcription factors (TFs). This study provides insight into the molecular mechanism of the drought stress response of *C. vietnamensis* and provides a theoretical basis for the development and cultivation of new drought-resistant cultivars.

## 1. Introduction

Drought stress is an abiotic stress that has a considerable impact on agricultural production and industrial development worldwide, seriously affecting the yield and quality of crops [1]. Approximately 40% of global land is in arid and semiarid regions, and half of China’s land is in arid and semiarid regions [2]. The increasing global population and climate change pose a threat to water resources, leading to droughts that may become increasingly serious in the future, severely threatening global grain and oil crop yields [3,4]. Correspondingly, population growth leads to an increase in the demand for grain and oil. Therefore, it is particularly necessary to understand the physiological and molecular mechanisms of important grain and oil crops in response to drought stress and to cultivate drought-resistant cultivars with a high yield and quality.

*Camellia vietnamensis* Huang (*C. vietnamensis*), belonging to the genus *Camellia* in the Theaceae family, is a woody oil tree species growing in southern China [5]. The oil extracted from the seeds of *C. vietnamensis* is called *Camellia* spp. and contains plenty of nutrients that are beneficial to the human body, such as unsaturated fatty acids, vitamins and squalene. This oil can increase immunity, inhibit tumors, reduce cholesterol and have other effects, and the long-term consumption of this oil can also play a role in prolonging life, known as “oriental olive oil” [6]. Hainan Island is located at the southernmost tip of China. *C. vietnamensis* on the island has been considered an independent population after its long-term geographical isolation [7]. The unique island conditions and genetic characteristics have formed rich and unique *C. vietnamensis* cultivars, making the quality of tea oil produced by *C. vietnamensis* in Hainan Island far superior to other cultivars planted in the mainland [8]. At the same time, the byproducts of *C. vietnamensis* also have great practical value in the industry, biology and medicine [9]. Although *C. vietnamensis* has certain drought-resistance properties, the extreme environment caused by water shortages is the main limiting factor for the growth of *C. vietnamensis* [1]. Drought occurring during the growth of *C. vietnamensis* hinders the accumulation of nutrients in *C. vietnamensis* fruit, causes the yellowing of leaves and reduces photosynthesis and fruit abscission, which leads to a decline in *C. vietnamensis* quality and yield [10]. Therefore, to improve the yield and quality of *C. vietnamensis*, the research on its drought resistance is essential.

In the long-term evolution of plants, many complex physiological, biochemical and molecular regulatory mechanisms have formed to help plants survive under drought stress. Different plants have different mechanisms of drought resistance to drought stress. In terms of physiology and biochemistry, plants absorb water by slowing the growth rate and reducing the yield [11], improving the properties of roots and increasing the root–shoot ratio [12]. Additionally, the stomatal aperture of plants continuously decreases, which reduces the conductivity of the stomata, slows photosynthesis and destroys photosynthetic pigments [13,14]. At the same time, plants can adapt to drought stress by activating respiration and complex hormone-signal regulatory networks [15], increasing osmoregulatory substances and enhancing antioxidant enzyme activities to maintain a stable balance between light capture and energy use [16,17]. In terms of molecular regulation, plants survive drought stress by regulating a series of stress-related gene expressions and signal transduction pathways [18]. Casaretto et al. observed that the overexpression of *OsMYB55* in maize can improve tolerance to drought [19]. Wen et al. identified 2917 genes involved in the drought stress response in *Betula* and observed that the transcription factors *BpERF2* and *BpMYB102* could regulate a series of drought-tolerance genes in *Betula* platyphyllum to improve drought tolerance [20]. Chang et al. found in rice cultivar KY131 that the combined overexpression of abscisic acid signaling pathway protein kinase (*SAPK6*) and bZIP transcription factor (*OsbZIP46CA1*) can further enhance the resistance to drought stress [21].

Recently, transcriptome sequencing technology has been widely used in the study of plant-drought-resistance mechanisms. For example, LI et al. conducted RNA sequencing on *Lilium davidii* var. *unicolor*, and found that drought resistance could be improved by regulating photosynthesis; increasing osmotic pressure; and activating the circadian rhythm, signal transduction, fatty acid metabolism and phenylalanine and flavonoid metabolism pathways so that it could resist drought stress [22]. Zhang et al. conducted the RNA sequencing of two glutinous millet cultivars and found that the drought resistance of glutinous millet could be improved by regulating the signal transduction pathway of jasmonate so that it could adapt to drought stress [23]. Zhao et al. conducted RNA sequencing on Jerusalem artichoke seedlings and found that they responded to drought stress by regulating the biosynthesis of secondary metabolites, phenylpropanoid biosynthesis and flavonoid biosynthesis, thereby improving their drought resistance [24]. This result indicates that transcriptome sequencing technology is an efficient method to obtain transcriptome data [25,26]. However, most of the previous studies on the drought resistance of *C. vietnamensis* focus on morphological and physiological responses [27]. In recent years, some researchers have also reported the transcriptional characteristics of *C. vietnamensis* [1]. However, the response and adaptation mechanism to drought stress has rarely been studied, especially among different cultivars having different tolerance levels. In this study, drought-tolerant (HD1) and drought-sensitive (WH1) cultivars are used as experimental materials, and physiological and transcriptome comparison analyses are conducted under PEG-simulated drought stress for 0 d, 3 d and 6 d at the seedling stage to reveal the physiological and molecular mechanisms of *C. vietnamensis* response to drought stress.

## 2. Results

### 2.1. Comparative Analysis of Morphological and Physiological Characteristics of HD1 and WH1 C. vietnamensis Seedlings under PEG Stress

**Figure 1:**
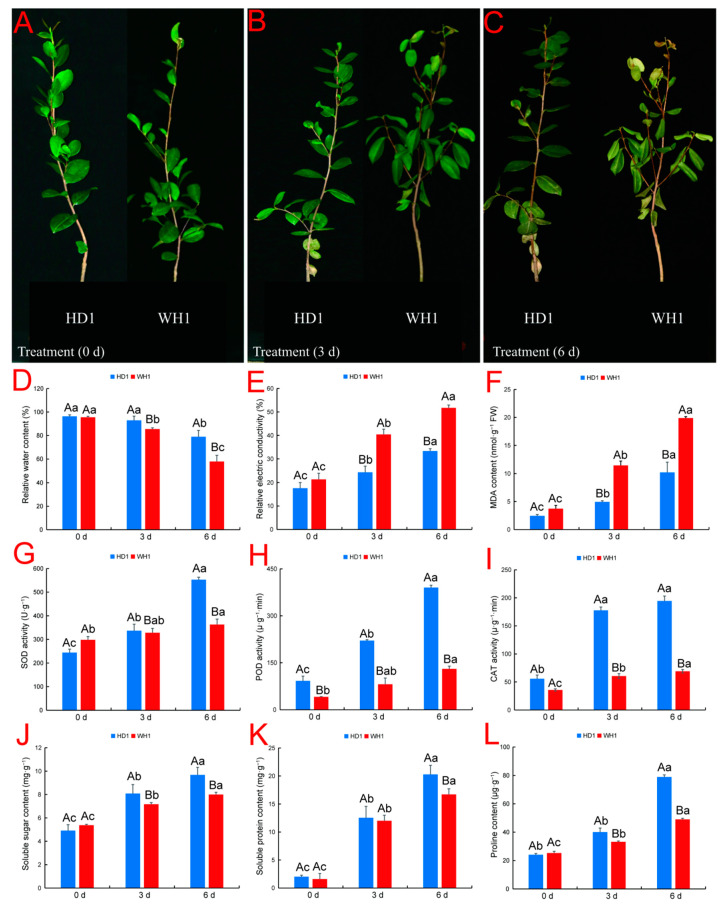
Phenotypic and physiological responses of drought-tolerant cultivar HD1 and drought-sensitive cultivar WH1. (**A**): Unstressed plants (0 d); (**B**): PEG stress treatment for three days (3 d); (**C**): PEG stress treatment for six days (6 d); (**D**): leaf relative water content; (**E**): relative electric conductivity; (**F**): MDA content; (**G**): superoxide dismutase (SOD) activity; (**H**): peroxidase (POD) activity; (**I**): catalase (CAT) activity (**J**): soluble sugar (SS) content (**K**): soluble protein (SP) content (**L**): free proline (Pro) content. All data are shown as the mean standard error. Different capital letters represent significant differences between cultivars; different lowercase letters indicate significant differences between treatments (Duncan’s test; *p <* 0.05).

The damage degree of PEG-simulated drought stress to different *C. vietnamensis* cultivars was significantly different. Without PEG stress treatment (0 d), the two cultivars of *C. vietnamensis* had no significant changes in morphology (Figure 1A). However, when treated with PEG for 3 d and 6 d, the leaves of WH1 showed significant symptoms, such as leaf drooping, curling and wilting, with increasing stress time, while HD1 plants showed only a mild stress phenotype (Figure 1B,C). The physiological characteristics of two *C. vietnamensis* cultivars were detected, and it was found that under PEG stress, with increasing stress time, the leaf RWC decreased, and relative electrical conductivity (REC), MDA content, antioxidant enzyme activities (superoxide dismutase (SOD), peroxidase (POD) and catalase (CAT)) and osmoregulatory substance contents (soluble sugars (SS), soluble protein (SP) and proline (Pro)) increased (Figure 1D–L). At 0 d of stress treatment, compared with 3 d and 6 d of stress treatment, the RWC of HD1 leaves decreased by 3.7% and 22.1%, respectively, and that of WH1 leaves decreased by 11.8% and 65.1%, respectively. The drought-sensitive cultivar WH1 lost water faster than the drought-tolerant cultivar HD1. Under the drought stress simulated by PEG, the REC and MDA contents of HD1 were always lower than those of WH1, while the activities of antioxidant enzymes (SOD, POD and CAT) and the contents of osmoregulatory substances (SS, SP and Pro) were always higher than those of WH1. Specifically, the fold change of REC and MDA content of WH1 with respect to HD1 at 6 days were 1.6 and 1.9, respectively. On the 6th day of treatment, the fold change of SOD, POD and CAT activities and the SS, SP and Pro contents of WH1 with respect to HD1 were 0.7, 0.3, 0.4, 0.8, 0.8 and 0.6, respectively. These results show that PEG-simulated drought stress may lead to the accumulation of reactive oxygen species (ROS) and the enhancement of membrane lipid peroxidation in the drought-sensitive cultivar WH1 and cause the plant to suffer more serious stress damage.

### 2.2. Overview of Leaf Transcriptome Sequencing Results of C. vietnamensis under Stress by PEG

The leaves of two *C. vietnamensis* cultivars (WH1 and HD1) at 3 d and 6 d after PEG stress treatment were used as the treatment group, and the leaves at 0 d were used as the control group. A cDNA library of 18 *C. vietnamensis* leaves was constructed. A total of 117.97 GB of clean data was obtained after RNA-seq. The effective data amount of each sample was distributed in 5.99–6.88 GB, the Q30 base was distributed in 92.18–92.53% and the average GC content was 45.37% (Table S1). By comparing the reads to the reference genome, approximately 83.92% and 83.91% of the read data of HD1 and WH1 leaves could be mapped to the reference genome of *C. vietnamensis*, and approximately 72.46% and 72.49% of HD1 and WH1 read data were uniquely mapped to the reference genome, respectively (Table S1). Principal component analysis (PCA) and FRKM boxplots showed that PEG-simulated drought stress resulted in significant changes in gene expression in *C. vietnamensis* leaves (Figure S1). The relevant heatmap and clustering tree diagram indicate that the experimental reliability of the subsequent analysis and selection of sample materials are reasonable (Figure S2).

### 2.3. Analysis of DEGs between HD1 and WH1 Cultivars

**Figure 2:**
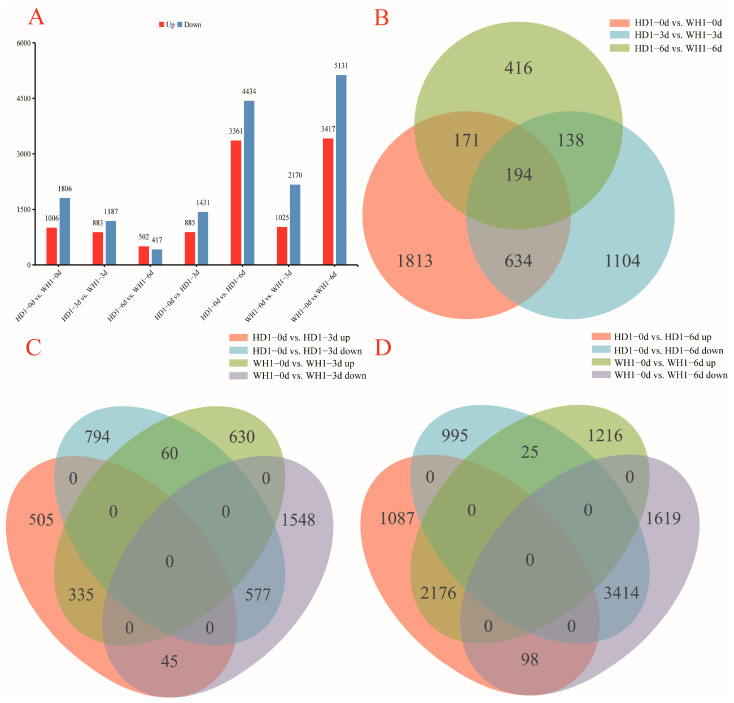
Differentially expressed genes (DEGs) in HD1 and WH1 under PEG stress. The selection of DEGs is based on the cutoff value of *p*-value < 0.05 and | log2FC | ≥ 2. (**A**): The total number of differentially expressed genes in HD1 and WH1 under PEG stress (**B**): The Venn diagram shows the comparison data between HD1-0d and WH1-0d, HD1-3d and WH1-3d, and HD1-6d and WH1-6d. (**C**): Venn diagram shows the comparison of DEGs expressed by two *C. vietnamensis* cultivars under PEG stress for 3 d. (**D**): Venn diagram shows the comparison of DEGs expressed by two *C. vietnamensis* cultivars under PEG stress for 6 d.

The two cultivars obtained a total of 2812 DEGs by comparison with their control samples collected at 0 d. A total of 2070 and 919 DEGs were identified in HD1-3d vs. WH1-3d and HD1-6d vs. WH1-6d, respectively (Figure 2A). A total of 194 DEGs were found in the two cultivars (Figure 2B).

The 2316 DEGs (including 885 upregulated and 1431 downregulated genes) and 3195 DEGs (including 1025 upregulated and 2170 downregulated genes) were obtained in HD1-0 d vs. HD1-3 d and WH1-0 d vs. WH1-3 d, respectively. In HD1-0d vs. HD1-6d and WH1-0d vs. WH1-6d, 7795 DEGs (including 3361 upregulated and 4434 downregulated genes) and 8548 DEGs (including 3417 upregulated and 5131 downregulated genes) were identified (Figure 2A). With the extension of stress time, the number of DEGs gradually increased. Compared with HD1, the fold change of the WH1 DEGs were 1.4 and 1.1 at 3 d and 6 d of stress treatment, respectively (Figure 2A). The results show that there are more regulated genes in drought-sensitive than in drought-tolerant cultivars under PEG stress.

By comparing and analyzing the differences of 0 d vs. 3 d of the two cultivars, 1299 genes (505 upregulated and 794 downregulated genes) were specifically expressed in HD1, and 2178 genes (630 upregulated and 1548 downregulated genes) were specifically expressed in WH1. There were 335 common upregulated and 577 common downregulated genes in the two cultivars. There were 45 upregulated and 60 downregulated genes in HD1, while the expression trend was the opposite in WH1 (Figure 2C). By comparing and analyzing the differences at 0 d vs. 6 d between the two cultivars, we found that 2082 genes (1087 upregulated and 995 downregulated genes) were specifically expressed in HD1, and 2835 genes (1216 upregulated and 1619 downregulated genes) were specifically expressed in WH1. There were 2176 common upregulated and 3414 common downregulated genes in the two cultivars. There were 98 upregulated and 25 downregulated genes in HD1, while the expression trend was the opposite in WH1 (Figure 2D).

**Figure 3:**
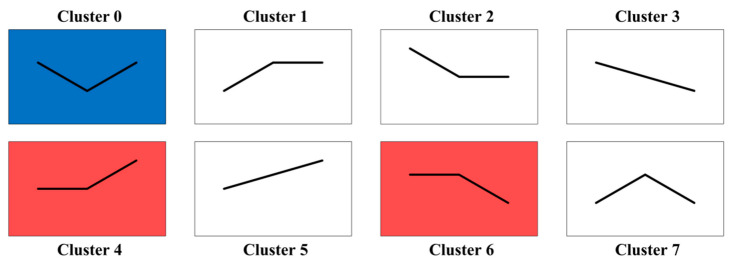
Expression patterns of the DEGs under the treatments in the two *C. vietnamensis* cultivars on the basis of STEM analysis.

Cluster analysis was performed on DEGs generated between different treatments of two cultivars using STEM software. The results show that these DEGs can be divided into eight expression patterns (Figure 3, Table S2). According to the expression trend analysis, the gene expression levels of the two *C. vietnamensis* cultivars under PEG stress were significantly different with increasing stress duration. In HD1, DEGs were mainly enriched in clusters 4 and 6, but in WH1, DEGs were significantly enriched in clusters 0, 4 and 6. Clusters 4 and 6 contained 2437 and 4990 DEGs in HD1, while clusters 0, 4 and 6 contained 2062, 2272 and 4668 DEGs in WH1, respectively.

### 2.4. GO- and KEGG-Enrichment Analyses

**Figure 4:**
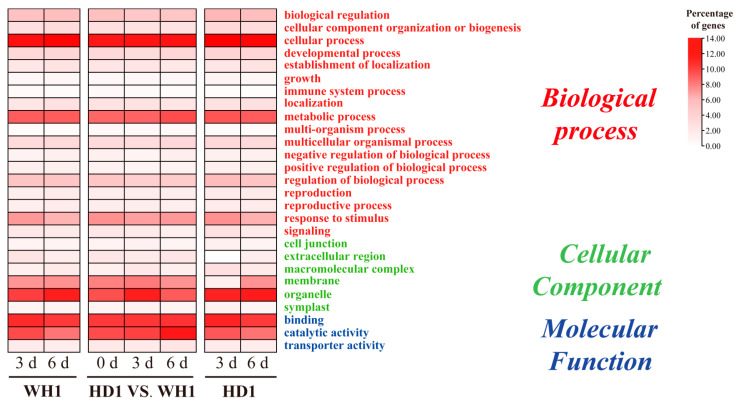
Functional annotations based on *C. vietnamensis* GO term analysis. The results are classified into three major GO classes, namely, biological process (BP), cellular component (CC) and molecular function (MF). Blocks from left to right indicate the GO term from comparisons of WH1-0d vs. WH1-3d, WH1-0d vs. WH1-6d, HD1-0d vs. WH1-0d, HD1-3d vs. WH1-3d, HD1-6d vs. WH1-6d, HD1-0d vs. HD1-3d, and HD1-0d vs. HD1-6d.

To further explore the molecular response mechanism of the two *C. vietnamensis* cultivars to PEG-simulated drought stress, GO- and KEGG-enrichment analyses were performed on the DEGs generated by pairwise comparisons at different treatment times to reveal the role of PEG in simulating drought-stress-response genes. According to the GO terminology analysis, dynamic distribution maps of 27 significantly enriched GO terms were obtained (Figure 4, Table S3). In both cultivars, genes associated with GO classes, such as “metabolic processes”, “stimulus response”, “cellular processes” and “biological regulation”, were highly abundant in biological processes (BPs). Genes associated with GO classes, such as “binding”, “catalytic activity” and “transport activity”, were highly abundant in molecular function (MF). In terms of cellular component (CC), genes were highly abundant in GO categories, such as “membrane”, “cell part” and “cell junction”. Cell architecture was seriously impacted in WH1 during 3d stress treatment, as evidenced by BP GO in “cellular process” and “response to stimulus”. Furthermore, cell architecture-related CC, such as “membrane”, “cell part” and “cell junction”, were highly enriched in the drought-sensitive cultivars (Figure 4). Upon stress treatment for 6d, the two *C. vietnamensis* cultivars were enriched in “stimulation response” and “metabolic process” in BP GO terms, “membrane” and “organelle” in CC GO terms and “catalytic activity” and “binding” in MF GO terms (Figure 4). These results indicate that drought stress activates the defense-related genes, improves the metabolic process and increases the antioxidant enzyme activity of *C. vietnamensis*.

**Figure 5:**
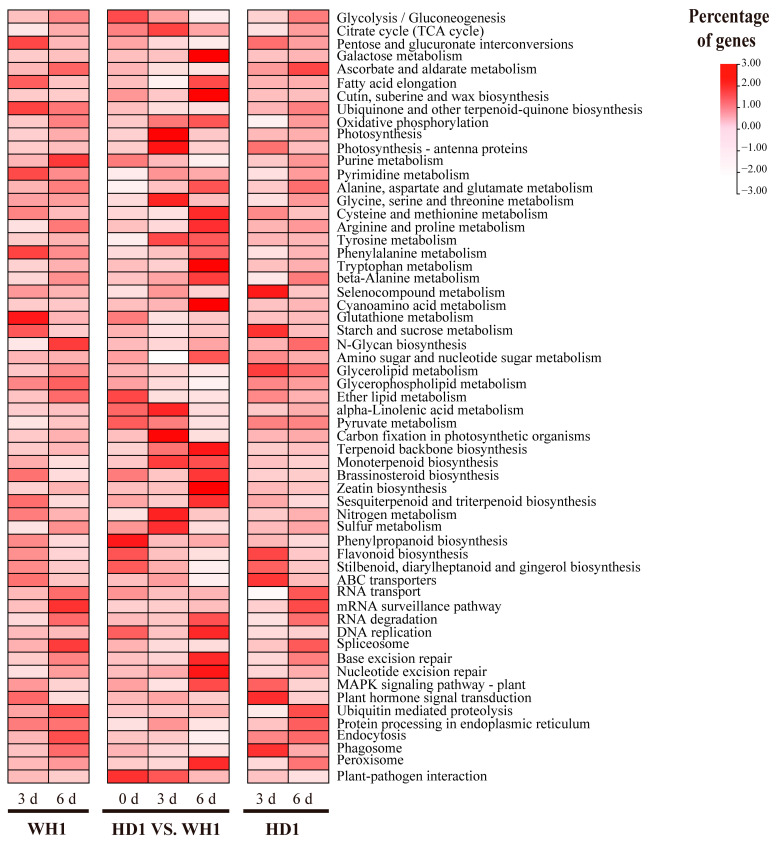
KEGG pathway-enrichment analysis based on *C. vietnamensis*. Blocks from left to right indicate the KEGG pathway-enrichment analysis from comparisons of WH1-0d vs. WH1-3d, WH1-0d vs. WH1-6d, HD1-0d vs. WH1-0d, HD1-3d vs. WH1-3d, HD1-6d vs. WH1-6d, HD1-0d vs. HD1-3d, and HD1-0d vs. HD1-6d.

According to KEGG analysis, DEGs identified in HD1-0d vs. WH1-0d, HD1-3d vs. WH1-3d and HD1-6d vs. WH1-6d were enriched in 105, 98 and 75 pathways, respectively (Table S4). There were 63 common KEGG pathways for the three pairwise comparisons. HD1-0d vs. HD1-3d and HD1-0d vs. HD1-6d were enriched in 92 and 122 KEGG pathways (Table S4), respectively. There were 92 common KEGG pathways. DEGs of WH1-0d vs. WH1-3d and WH1-0d vs. WH1-6d were enriched in 103 and 122 KEGG pathways (Table S4), respectively. There were 102 common KEGG pathways. The KEGG pathways enriched in the above three groups were comprehensively analyzed and collectively enriched in 59 pathways (Figure 5, Table S5). Carbohydrate metabolism, lipid metabolism, terpenoid and polyketone metabolism and other secondary metabolite biosyntheses are the largest part of the metabolites. RNA transport- and spliceosome-related genes were the most important genes in genetic information processing. Endocytosis- and peroxisome-related genes were the most abundant in cell processes. ABC transporters, plant-hormone signal transduction and the MAPK signaling pathway are abundant in environmental information processing. Amino acids related to plant-pathogen interactions were the most abundant in biological systems.

### 2.5. Different Expression Patterns of Genes Related to Phenylpropanoid Biosynthesis in Two C. vietnamensis Cultivars under PEG Stress

**Figure 6:**
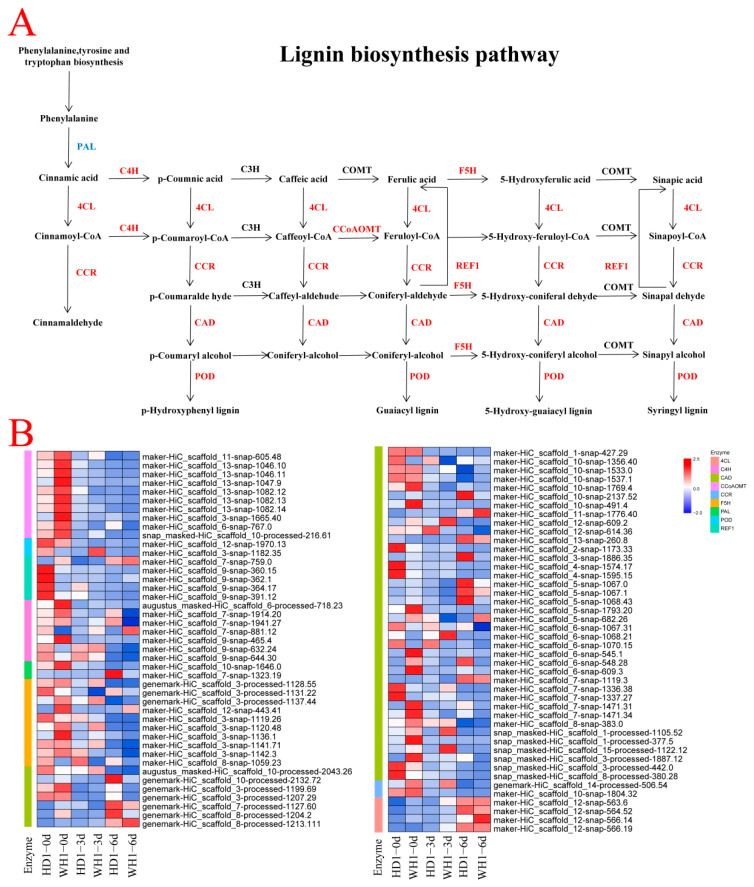
Expression analysis of DEGs enriched in the lignin biosynthesis pathway. (**A**): DEGs enriched in the lignin biosynthesis pathway in *C. vietnamensis* leaves. (**B**): Heatmap of lignin biosynthesis-related gene expression. PAL, phenylalanine ammonia-lyase; C4H, trans-cinnamate 4-monooxygenase; 4CL, 4-coumaric acid: CoA ligase; CCR, cinnamoyl CoA reductase; CAD, cinnamyl alcohol dehydrogenase; POD, peroxidase; COMT, caffeate 3-O-methyltransferase; CCoAOMT, caffeoyl-CoA O-methyltransferase; F5H, ferulate 5-hydroxylase; REF1, coniferyl-aldehyde dehydrogenase. Values are the average FPKM value of each sample in each group. Genes in red or blue refer to up- or downregulation responding to drought, respectively.

Transcriptome analysis showed that 87 DEGs were enriched in the lignin biosynthesis pathway, including PAL (10), C4H (2), CCR (5), 4CL (7), F5H (2), CAD (10), POD (45), CCoAOMT (2) and REF1 (4) (Figure 6, Table S6). Subsequently, we analyzed the differential expressions of key enzyme genes in the lignin biosynthesis pathway of the two *C. vietnamensis cultivars*. We focused on the comparison analysis of HD1-3d vs. WH1-3d and HD1-6d vs. WH1-6d time points. In the lignin biosynthesis pathway, compared with WH1, seven out of ten genes involved in the regulation of the PAL enzyme in HD1 were upregulated under the 3 d and 6 d treatments. After 3 d of stress treatment, four of ten CAD enzyme genes were upregulated, and six were downregulated in HD1; after 6 d of treatment, six genes were upregulated and four genes were downregulated. The upregulated expressions of POD (maker_HiC-scaffold_5-snap-1068.43, maker_HiC-scaffold_7-snap-1471.34 and maker_HiC-scaffold_6-snap-548.28) and 4CL (maker_HiC-scaffold_7-snap-1941.27) were observed in the leaves of HD1 *C. vietnamensis* under PEG stress. The transcript of POD (maker_HiC-scaffold_5-snap-1068.43 and maker_HiC-scaffold_6-snap-548.28) was enhanced 2.02 times and 4.03 times when HD1 was treated for 3 d, and strongly enhanced 2.90 times and 27.4 times when HD1 was treated for 6 d, but decreased in WH1, suggesting that there were different POD gene expression patterns between HD1 and WH1 in response to drought stress.

### 2.6. Different Expression Patterns of Genes Related to Flavonoid Biosynthesis in Two C. vietnamensis Cultivars under PEG Stress

**Figure 7:**
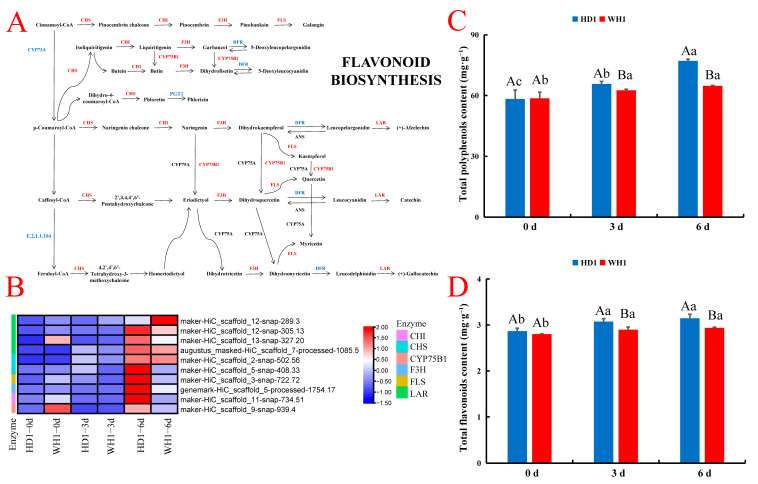
Expression analysis of DEGs enriched in the flavonoid biosynthesis pathway. (**A**): DEGs enriched in the flavonoid biosynthesis pathway in *C. vietnamensis* leaves. (**B**): Heatmap of flavonoid biosynthesis-related gene expression. CHI, chalcone isomerase; CHS, chalcone synthase; CYP75B1, flavonoid 3′-monooxygenase; F3H, flavanone 3-hydroxylase; FLS, flavonol synthase; LAR, leucoanthocyanidin reductase. Values are the average FPKM value of each sample in each group. Genes in red or blue refer to up- or downregulation responding to drought, respectively. (**C**). Total polyphenol content of drought-tolerant cultivar HD1 and drought-sensitive cultivar WH1. (**D**). Total flavonoid content of the drought-tolerant cultivar HD1 and drought-sensitive cultivar WH1. All data are shown as the mean standard error. Different capital letters represent significant differences between cultivars; different lowercase letters indicate significant differences between treatments (Duncan’s test; *p* < 0.05).

By comparing the expression patterns of HD1- and WH1-related genes in the flavonoid biosynthesis pathway, we identified ten key regulatory DEGs, including LAR (4), CHS (2), FLS (1), F3H (1), CHI (1) and CYP75B1 (1) (Figure 7, Table S7). Most of the genes related to the flavonoid biosynthesis pathway were inhibited at 3 d of treatment, and most of the genes resumed upregulation in both cultivars under PEG-induced drought stress after 6 d of stress. In HD1, four genes regulating LAR and two genes regulating CHS were upregulated at 3 d and 6 d compared with 0 d. In WH1, compared with 0 d, four genes that regulate the LAR enzyme were upregulated by two and downregulated by two at 3 d of treatment and upregulated by three and downregulated by one at 6 d of treatment; two genes regulating CHS enzymes were upregulated by one and downregulated by one at 3 d of treatment, and two were upregulated at 6 d of treatment. Compared with WH1, in HD1, two of the four genes regulating LAR were upregulated and two were downregulated at 3 d of treatment, and three were upregulated and one was downregulated at 6 d of treatment. The two genes regulating CHS were upregulated after 3 d and 6 d of treatment. Therefore, compared with WH1, most of the genes encoding the key enzymes LAR, CHS, FLS, F3H, CHI and CYP75B1 were upregulated in HD1 at 3 d and 6 d of stress. These results indicate that HD1 can activate the upregulated expression of more key genes in the flavonoid biosynthesis pathway to improve the drought resistance of plants under PEG stress. To further illustrate the response of PEG-simulated drought stress to the flavonoid biosynthesis pathway of two *C. vietnamensis* cultivars, we determined the contents of total flavonoids (Flas) and total polyphenols (Pols) in two *C. vietnamensis* cultivars. We found that PEG-induced water stress significantly increased the accumulation of Fla and Pol in the leaves of both *C. vietnamensis* cultivars (Figure 7C,D). These results suggest that the excessive accumulation of flavonoids may be an adaptive mechanism of *C. vietnamensis* resistance to drought stress.

### 2.7. Different Expression Patterns of Hormone Signal Transduction-Related Genes in Two C. vietnamensis Cultivars under PEG Stress

**Figure 8:**
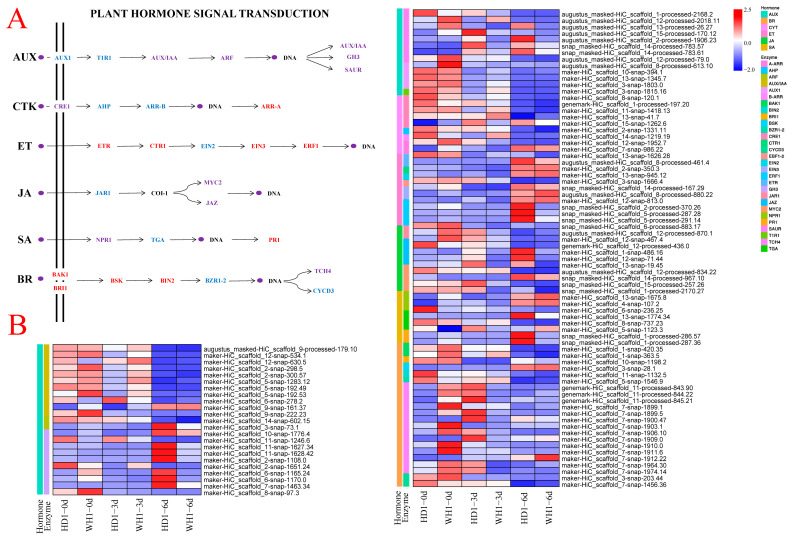
Expression analysis of DEGs enriched in the plant-hormone signal transduction pathway. (**A**): DEGs enriched in the plant-hormone signal transduction pathway in *C. vietnamensis* leaves. AUX, auxin signal; CTK, cytokinin signal; ET, ethylene signal; JA, jasmonic acid signal; SA, salicylic acid; BR, brassinosteroid signal. Genes in red or blue refer to up- or downregulation responding to drought, respectively. Genes in purple refer to both up- and downregulation in response to drought. (**B**): Heatmap of plant-hormone signal transduction-related gene expression. Values are the average FPKM value of each sample in each group.

In the plant-hormone signal transduction pathway, AUX, CTK, ET, JA, SA and BR were found to be involved in the response of the two *C. vietnamensis* cultivars to PEG-induced drought stress (Figure 8, Table S8). There were significant differences between HD1 and WH1 in several key enzymes enriched in the abovementioned signaling pathways. We focused on the comparison of HD1-3d vs. WH1-3d and HD1-6d vs. WH1-6d time points. In the AUX signaling pathway, the differentially expressed genes were mainly enriched in AUX/IAA, ARF, GH3, SAUR, AUX1 and T1R1. Compared with WH1, in HD1, nine genes encoding the auxin-inducing protein AUX/IAA were downregulated, and two genes were upregulated under the 3 d and 6 d treatments. Two upregulated genes encoding ARF, six upregulated genes encoding CH3 and seven upregulated genes encoding SAUR were found after HD1 treatment for 3 d, and nine upregulated genes encoding CH3 and SAUR were found after HD1 treatment for 6 d. The expression of three genes encoding AUX1 was inhibited by HD1 at 3 d and 6 d. In the CTK signaling pathway, DEGs were mainly enriched in CRE1, AHP, B-ARR and A-ARR. Compared with WH1, the four genes involved in encoding CRE1 were suppressed at 3 d and upregulated at 6 d of HD1 treatment. Maker-HiC-scaffold-14-snap-1219.19 encoding AHP was inhibited by HD1 at 3 d and 6 d, respectively. The genes encoding A-ARR and B-ARR were upregulated after 6 d of HD1 treatment. In the ET signaling pathway, the DEGs were mainly enriched in ETR, CTR1, EIN2, EBF1-2, EIN3 and ERF1. Compared with WH1, the genes encoding CTR1 and EIN2 in HD1 were upregulated under the 3 d treatment and downregulated at 6 d of treatment. The genes involved in the regulation of EBF1-2 and ERF1 were upregulated at 3 d and 6 d. In the JA signaling pathway, DEGs were mainly enriched in JAR1, JAZ and MYC2. Compared with that in WH1, the JAR1 gene was downregulated in HD1 after 6 d of treatment. The gene encoding the jasmonate ZIM domain protein (JAZ) was downregulated in both the 3 d and 6 d treatments compared with WH1. Compared with WH1, three of the four genes in MYC2 were upregulated and one was downregulated under 3 d of treatment, while all of them were upregulated under 6 d of treatment. In the SA signaling pathway, the DEGs were mainly enriched in NPR1, TGA and PR1. Compared to WH1, the NPR1 (maker-HiC_scaffold_6-snap-236.25), TGA (maker-HiC_scaffold_13-snap-1774.34) and PR1 (snap_masked-HiC_scaffold_1-processed-286.57) genes were upregulated by HD1 treatment for 3 d and 6 d. The PR1 (snap_masked- hic_scaffolD_1-processed-286.57 and SNAP_masked–hic_SCAFFold_1-processed-287.36) gene in HD1 was treated with PEG for 6 d. Compared with the drought-sensitive cultivar, the gene expression increased 21.11 and 10.45 times. In the BR signaling pathway, the DEGs were mainly enriched in BAK1, BRI1, BSK, BIN2, BZR1-2, TCH4 and CYCD3. In HD1, the genes encoding BAK1 and BRI1 were inhibited at 3 d and upregulated at 6 d compared with WH1. The expression of genes involved in the regulation of BIN2 was upregulated at 3 d and 6 d.

### 2.8. TF Prediction

**Figure 9:**
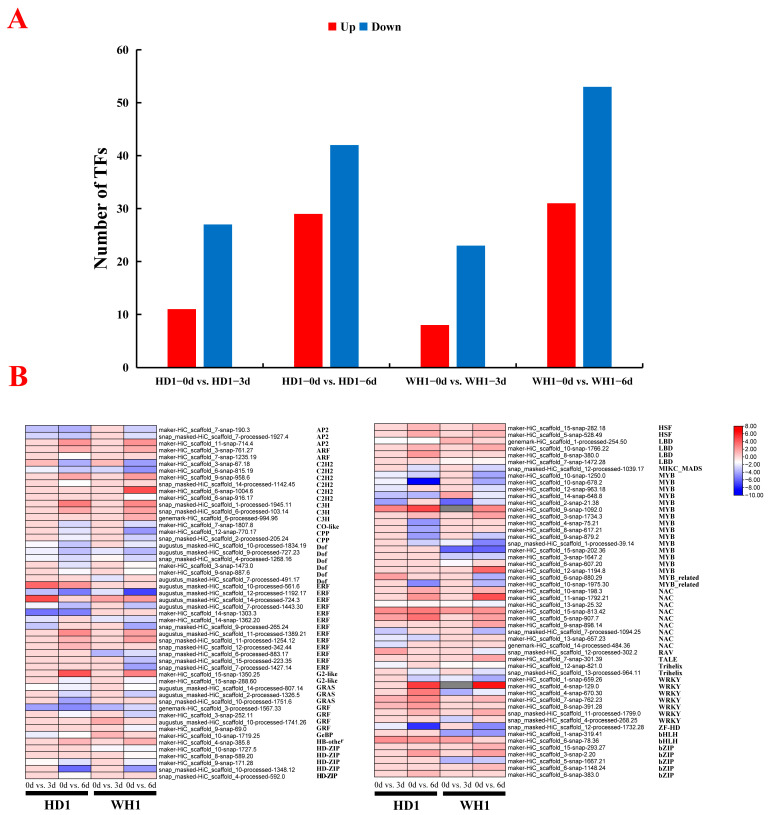
Transcription factor (TF) analysis of *C. vietnamensis* leaves under PEG stress. (**A**): The number of TF of two *C. vietnamensis* cultivars compared between different groups. (**B**): Heatmap of TF-related gene expression. Blocks from left to right indicate the TF from comparisons of HD1-0d vs. HD1-3d, HD1-0d vs. HD1-6d, WH1-0d vs. WH1-3d, and WH1-0d vs. WH1-6d. The value is the log2(fold change) value of each sample in each group.

A total of 191 genes were identified and annotated into 35 TF gene families (Figure 9), with significant changes in transcription factor families, including C2H2, Dof, HD-ZIP, ERF, MYB, NAC, WRKY, C3H and HSF (Figure S3). We analyzed the number of differentially expressed TFs between different groups in the two cultivars. There were 11 upregulated TFs and 27 downregulated TFs in drought-tolerant cultivars and 8 upregulated TFs and 23 downregulated TFs in drought-sensitive cultivars. However, at 6 d of PEG stress, the number of differentially expressed TFs was the highest, with 29 upregulated and 42 downregulated in drought-tolerant cultivars and 31 upregulated and 53 downregulated in drought-sensitive cultivars. This indicated that the number of differentially expressed TFs increased with prolonged PEG stress time. The DEGs encoding WRKY, ERF, C3H, HSF and NAC TFs were mostly upregulated in HD1. Most DEGs encoding MYB, HD-zip, AP2, C2H2 and Dof TFs were downregulated in HD1. The DEGs encoding C3H, HSF and NAC TFs were mostly upregulated in WH1. Most DEGs encoding WRKY, HD-zip, MYB, ERF, C2H2, AP2 and Dof TFs were downregulated in WH1.

### 2.9. RNA-seq Expression Level Was Verified by qRT-PCR

**Figure 10:**
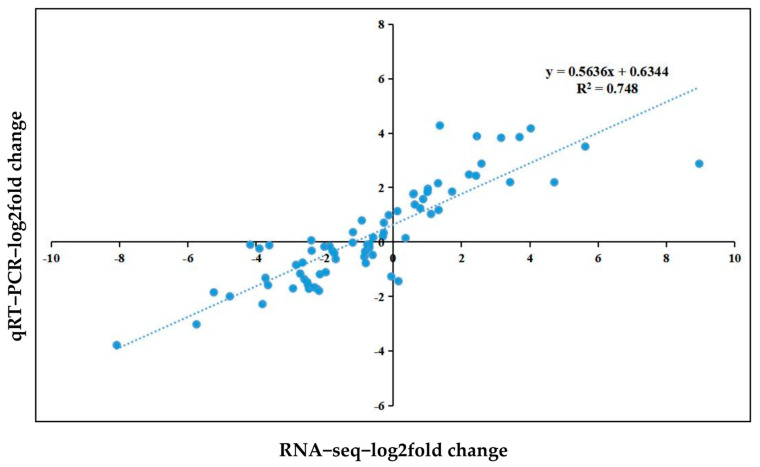
Correlation analysis between RNA-seq and qRT-PCR methods. Log2(fold change) values of RNA-seq data (x axis) are plotted against log2(fold change) values of qRT-PCR (y axis) data.

A total of 18 DEGs related to lignin biosynthesis were screened and verified by qRT-PCR. RNA-seq and qRT-PCR data showed that the expression levels of maker-HiC_scaffold_7-snap-759.0, maker-HiC_scaffold_12-snap-563.6 and maker-HiC_scaffold_12-snap-566.19 continuously increased with increasing stress duration. The expression levels of maker-HiC_scaffold_11-snap-605.48, maker-HiC_scaffold_9-snap-465.4, maker-HiC_scaffold_10-snap-1646.0, maker-HiC_scaffold_4-snap-1574.17 and maker-HiC_scaffold_10-snap-1804.32 continuously decreased with increasing stress duration. The expression pattern of DEGs obtained by qRT-PCR was highly consistent with the results of RNA-seq (Figure S4). The results show that there is a significant positive correlation between the RNA-seq and qRT-PCR data, and the determination coefficient (R2) is 0.748 (Figure 10). The above data results indicate that the obtained RNA-seq data are reliable.

## 3. Discussion

*C. vietnamensis* is an economically important woody oil species in South China and plays an important role in agriculture, industry and medical services. However, the resistance ability of *C. vietnamensis* to drought stress is relatively weak. The study of the response of *C. vietnamensis* to drought stress can provide a theoretical basis for the development of new cultivars of *C. vietnamensis* and improve the adaptability of other plants to drought stress. Different cultivars with different genetic backgrounds are very important for the improvement of new cultivars [28]. In this study, the physiological responses of leaves of two *C. vietnamensis* cultivars, HD1 and WH1, to drought stress can provide a valuable reference for the breeding of *C. vietnamensis* drought-tolerant cultivars.

### 3.1. Physiological Responses of Two C. vietnamensis Cultivars to Drought Stress

Physiological indexes showed that the drought-tolerant cultivar HD1 and drought-sensitive cultivar WH1 had significant differences under PEG stress. With the increase in PEG stress treatment time, the droop angle, curl and wilting degree of HD1 leaves were less than those of WH1 (Figure 1A–C). The degree of leaf curl under drought stress may be related to the ability of leaf water regulation potential and soil water absorption. Water is an important component for the survival of plant cells and for maintaining metabolic and physiological functions. The leaf structure of drought-resistant cultivars is more conducive to water conservation [29]. Under PEG treatment for 3 d and 6 d, the RWC of the drought-tolerant cultivar was higher than that of the drought-sensitive cultivar (Figure 1D). Liu and Waititu et al. found that the RWC of drought-tolerant cultivars was significantly higher than that of drought-sensitive cultivars [30,31]. Maintaining a relatively high RWC may play an important role in drought-tolerant cultivars to better complete various physiological and biochemical processes and metabolic reactions. The cytomembrane is the first part that senses the effects of drought stress, and its stability directly affects the metabolic process of plant leaves. The MDA content can reflect the damage degree of the membrane system. The lower the REC and MDA content in drought-tolerant cultivars (Figure 1E,F), the more effectively the membrane system can be protected, and the stability of the cell membrane can be maintained under PEG stress. Li et al. found that the REC and MDA contents of drought-tolerant watermelon cultivars were lower than those of drought-sensitive cultivars [32]. Osmotic regulation is one of the physiological responses of plants to adapt to drought [33]. In this study, drought-resistant cultivars accumulated more Pro, SP and SS than drought-sensitive cultivars (Figure 1J–L), activating osmotic regulation and thus changing the water potential of plants, which is related to improving the drought tolerance of drought-resistant cultivars [34]. Dong and Zenda et al. found that osmotic adjustment substances of drought-tolerant cultivars were significantly higher than those of drought-sensitive cultivars in studies on *C. vietnamensis* and maize [10,35]. Under drought stress, maintaining high RWC, osmotic regulatory substance content (Pro, SP and SS) and stability of the membrane system can help plants cope with water deficit caused by adversity [36]. Interestingly, the results of our physiological analysis are consistent with our RNA-seq results, and the response mechanisms of the two *C. vietnamensis* cultivars to PEG-simulated drought stress are different. Under PEG stress treatment for 3 d and 6 d, the number of DEGs in drought-tolerant cultivars was relatively low, and the number of TFs in drought-tolerant cultivars was higher than that in drought-sensitive cultivars at 3 d of stress treatment, while that in drought-sensitive cultivars was significantly higher than that in drought-resistant cultivars at 6 d of stress treatment (Figure 9a). In HD1, the higher RWC and osmotic regulatory substance contents (Pro, SP, SS) and the lower REC and MDA content may have caused them to change reactions at the cellular level to be relatively low and maintain highly stable levels of gene expression; therefore, compared with the WH1 transcriptome study reaction, the HD1 reaction was more limited. Transcriptomic analysis of *C. vietnamensis* [10] and *sorghum* [37] also found a similar trend, that is, drought-sensitive cultivars expressed more DEGs and TFs than drought-tolerant cultivars under drought stress.

### 3.2. Increasing the Antioxidant Activity of C. vietnamensis Seedlings Plays a Key Role in Resisting Drought Stress

We observed increased cell wall lignification in plants when subjected to environmental stress [38]. Lignin is an important component of xylem secondary cell walls, and drought stress can lead to the production of large amounts of lignin in plants, thus guaranteeing mechanical support and water transport [39]. Enhanced lignin biosynthesis is a key factor in plant adaptation to drought stress [40]. During drought stress, a large number of ROSs are rapidly induced [41]. In *Salvadora persica* L., drought stress-induced POD is very important for maintaining the ROS homeostatic state and increasing drought tolerance [42]. In this study, DEGs involved in the lignin biosynthesis pathway included PAL (10), C4H (2), CCR (5), 4CL (7), F5H (2), CAD (10), POD (45), CCoAOMT (2) and REF1 (4). The DEGs of the two *C. vietnamensis* cultivars were mainly enriched in the POD enzyme. Most of the genes regulating POD enzyme activity in drought-resistant cultivars were more highly expressed than those in drought-sensitive cultivars, thus helping drought-tolerant cultivars resist damage caused by ROS. Therefore, we determined the activities of three antioxidant enzymes (SOD, POD and CAT) to explore the protection ability of two *C. vietnamensis* cultivars against drought stress. The results show that the antioxidant enzyme activities of the two cultivars increase under drought stress, while the SOD, POD and CAT enzyme activities of the drought-tolerant cultivars are significantly higher than those of the drought-sensitive cultivars (Figure 1G–I), corresponding to the transcriptome results. The above research results show that, compared with drought-sensitive cultivars, drought-resistant cultivars have stronger antioxidant enzyme activity to resist adverse reactions caused by drought stress, thus regulating ROS homeostasis, reducing ROS damage and improving drought tolerance.

### 3.3. The Flavonoid Biosynthesis Pathway Plays an Important Role in Drought Tolerance of C. vietnamensis Seedlings

Flavonoids are a class of polyphenols with anti-free radical and antioxidant activities and are composed of flavonoids, flavonols, anthocyanins and isoflavones [43]. To cope with oxidative stress caused by drought stress, plants accumulate excessive secondary metabolites of flavonoids through the expression of key enzyme genes encoding the biosynthesis pathway of flavonoids, which may be the key response of plants to cope with drought stress [44]. CHS is a specific enzyme in the flavonoid biosynthesis pathway that catalyzes the combination of three molecules, malonyl-CoA, and one molecule, 4-coumaryl-CoA, to generate chalcone, which generates corresponding flavanones under the catalysis of CHI [45]. By transferring the CHS gene of okra into *Arabidopsis thaliana*, the flavonoid accumulation and abiotic stress tolerance of the plant were improved [46]. LAR catalyzed the formation of flavan-3-ol units by leucocyanidin and leucodelphinidin [47]. The overexpression of LAR increases the contents of catechin and epicatechin in plants [48]. The expression of the LAR gene in tea plants under drought stress can increase the content of catechin [49]. In this study, we found that four LAR genes, two CHS genes and one CHI gene were upregulated in drought-tolerant cultivars under drought stress compared with drought-sensitive cultivars. Therefore, we concluded that the expression of key enzymes in the flavonoid biosynthesis pathway can regulate the drought tolerance of *C. vietnamensis* under drought stress. To verify this inference, we also determined the contents of Fla and Pol in two *C. vietnamensis* cultivars (Figure 7C,D). The results show that drought-tolerant cultivars accumulated more Fla and Pol than drought-sensitive cultivars under PEG stress, and the increase in flavonoid secondary metabolites contributes to the improvement in the ROS scavenging ability of plants. Therefore, the flavonoid biosynthesis pathway may be a key factor in improving the drought tolerance of drought-tolerant cultivars.

### 3.4. Plant-Hormone Signal Transduction Plays a Crucial Role in Drought Tolerance of C. vietnamensis Seedlings, Especially AUX and BR

Drought stress can regulate the biosynthesis and signal transduction of endogenous hormones in plants [50]. In the plant-hormone signal transduction pathway, there were 97 significantly enriched DEGs involved in the auxin (AUX, 37), cytokinin (CTK, 9), ethylene (ET, 11), brassinosteroid (BR, 22), jasmonic acid (JA,10) and salicylic acid (SA,8) signaling pathways.

Auxin plays an important role in regulating plant growth, development and response to various stresses [51]. The TIR1/AFB-Aux/IAA-ARF signaling system is the main pathway in auxin signal transduction [52]. Aux/IAA protein plays an important role in auxin signal transduction as an auxin coreceptor and transcription suppressor [53]. In rice, the overexpression of the Aux/IAA gene *OsIAA6* improves drought resistance by regulating auxin biosynthesis-related genes [54]. In *sorghum*, *SbIAA1* is highly induced under drought treatment [55]. SAUR is an auxin early response gene family. The expression of the *SAUR39* gene in rice leads to the inhibition of root and leaf growth and an increase in sugar, abscisic acid and anthocyanin contents, thus changing the resistance of rice [56]. ARF can regulate and influence various stages of plant growth and development in auxin synthesis, such as embryo pattern establishment, vascular tissue formation, flower development and phototropism [57]. Posttranscriptional regulation of ARF expression through miR160 and miR165/166 can improve drought tolerance in *Arabidopsis* [58]. As an early auxin-response gene, CH3 plays an important role in the regulation of hormone homeostasis and the rapid response to abiotic stress. The *SbGH3* gene expressed at a low level in sorghum under natural conditions is highly induced under drought stress, and its products are involved in drought stress [59]. Our results show that compared with drought-sensitive cultivars, two AUX/IAA genes are upregulated and nine AUX/IAA genes are downregulated, SAUR and ARF genes were upregulated, and nine GH3 genes were upregulated and one GH3 gene was downregulated in drought-resistant cultivars. These results indicate that most of the auxin signal transduction genes in drought-resistant cultivars are modulated to participate in the regulation of drought resistance in *C. vietnamensis*. Brassinosteroid (BR) plays an important role in plant growth and development, such as cell elongation, senescence and coping with abiotic stress [60]. BR binds to the receptor kinase BRI1 in the extracellular region and, by binding BAK1, activates and regulates BSK, BIN2 and BZR to transmit BR signals [61,62]. In *A. thaliana*, the phosphorylation of *RD26* by BIN2 enhances drought resistance [63]. The overexpression of the BR biosynthesis gene *DWF4* in *Brassica napus* l. enhanced drought resistance [64]. Our study showed that BAK1, BRI1 and BIN2 were upregulated in drought-resistant cultivars compared with drought-sensitive cultivars, which may further activate the downstream gene expression of BR, thus improving the drought resistance of *C. vietnamensis*. Different regulation strategies of auxin and BR signal transduction pathways in drought-tolerant cultivars may play a key role in drought tolerance of *C. vietnamensis* seedlings.

Many studies have shown that ARR-B and ARR-A genes play key roles in CTK signal transduction, but there is a mutual regulatory effect between the two [65,66]. In this study, compared with those in drought-sensitive cultivars, ARR-B and ARR-A genes in drought-tolerant cultivars were upregulated after PEG treatment for 3 d and 6 d. EIN3 and ERF in the ET signaling pathway can promote drought tolerance in plants [67,68]. The overexpression of *AtERF019* can improve drought tolerance by delaying the growth and senescence of *Arabidopsis* [69]. Compared with drought-sensitive cultivars, the ERF1-2 and ERF1 genes in drought-tolerant cultivars were upregulated after PEG treatment for 3 d and 6 d. JAR1 is a JA amino synthase that activates JA by binding JA to Ile [70]. JAZ protein is a key regulator of jasmonic acid signaling [71]. MYC2 can activate downstream JA-mediated responses and the expression of most JAZ genes [72]. Some studies have shown that *OsJAZ1* expression can regulate JA and ABA signaling pathways to weaken drought resistance in rice [73]. *AtMYC2* is rapidly activated after traumatic response [74]. In this study, two JAR1, one JAZ and four MYC2 genes were downregulated in drought-tolerant cultivars compared with drought-sensitive cultivars under 6 d of drought stress. SA plays a crucial regulatory role in the plant defense response and abiotic stress [75]. Stomatal closure was observed through the accumulation of SA under drought stress [76]. In *Arabidopsis*, drought tolerance is enhanced by the expression of the SA-mediated PR gene to close stomata in *Arabidopsis* [77]. Similarly, our study found that PR1 gene expression in drought-tolerant cultivars was significantly increased compared with that in drought-sensitive cultivars after 6 d of PEG stress when drought-tolerant cultivars were subjected to drought stress. These results suggest that plant-hormone signals in drought-tolerant cultivars may be involved in the regulation of drought resistance in *C. vietnamensis* in a more complex way. Moreover, CTK, ET, JA and SA are endogenous hormones related to drought resistance in plants.

## 4. Materials and Methods

### 4.1. Plant Materials and Methods

In this experiment, two *C. vietnamensis* cultivars—the drought-tolerant cultivar ‘Haida 1’ (HD1) and the drought-sensitive cultivar ‘Wanhai 1’ (WH1)—with different drought tolerances were selected for research (Table 1). To avoid mixed provenances, all seedlings were cultivated by the Qionghai Wenquan Fafu *C. vietnamensis* Planting Farmers’ professional cooperative. The *C. vietnamensis* seedlings were planted in a monochromatic basin (11 cm high, 12.5 cm upper caliber, 9 cm lower caliber), and the cultivation substrate was a 1:3 vermiculite/peat soil mixture, which was placed in the agricultural base of Hainan University for unified maintenance.

After a slow seedling period of 3 months, *C. vietnamensis* seedlings with basically the same size and growth were selected for peeling off the soil and washing the roots. The washed *C. vietnamensis* seedlings were placed in half-strength Hoagland nutrient solution to slow the seedlings for 2d, and then transferred to the half-strength Hoagland nutrient solution containing 20% PEG (200 g·L^−1^ PEG; −0.6 MPa) [78] for stress treatment. PEG is a polymer penetrant often used to simulate drought stress experiments [10]. All experiments were conducted in an artificial climate chamber; the temperature was (27 ± 2) °C, the relative humidity was 75%–85%, the light intensity was 2000 lx, with 16 h daytime and 8h dark photoperiod treatments and ventilation was performed once in the morning and evening. The half-strength Hoagland nutrient solution with 20% PEG was replaced every 3 days. Samples were obtained at 0 d, 3 d and 6 d after PEG treatment. In each treatment, 3 *C. vietnamensis* seedlings were randomly selected as replicates, and samples were obtained from 3 to 7 fully developed mature leaves under the terminal bud. The obtained leaves were frozen in liquid nitrogen and stored at −80 °C for sample storage.

### 4.2. Measurement of Physiological, Biochemical and Secondary Metabolite Indexes

The leaf samples of two *C. vietnamensis* cultivars after PEG stress were used to determine the RWC of leaves, plant membrane permeability, antioxidant enzyme activity, osmoregulation substance and secondary metabolite contents.

The saturated water content method was used to determine the RWC of the leaves [79]. We quickly cut the fresh leaves and weighed them with an analytical balance for fresh weight (FR). Then, the weighed leaves were soaked in pure water for 24 h, the water on the leaf surface was dried with absorbent paper and the leaves were weighed and recorded. After soaking the leaves again for several hours, the leaves were removed and weighed until the results of the two weights were basically the same, which was recorded as saturated fresh weight (SA). The leaves were put into the oven at 105 °C for 10 min, then the temperature was adjusted to 80 °C and the leaves were dried to a constant weight. The leaves were weighed and the dry weight (DR) was recorded. The RWC was calculated using the following formula:

RWC(%)=(FR−DR)/(SA−DR)×100%

The REC of leaves was measured by the conductance meter method [80], with some modifications. Fresh leaves were obtained to wash surface stains with tap water and then washed three times with pure water. The surface moisture was aspirated with absorbent paper. The leaves were placed in a centrifuge tube, 10 mL of water was added and the mixture was left for 3 h. Then, the mixture was boiled in a thermostatic water bath at 100 °C for 20 min and cooled to room temperature with cold water, and the electrical conductivity (R2) was measured. The REC of the leaves was calculated using the following formula:

$$REC(\%)=\frac{R1}{R2}\times 100\%$$

The nitrogen blue tetrazole method was used to measure SOD activity (SOD-2-W Assay Kit, Suzhou, China). The visible spectrophotometric method was used to measure CAT and POD activities (CAT-2-W and POD-2-Y Assay Kit, Suzhou, China). The ninhydrin colorimetric method was used to measure the Pro content (PRO-2-Y Assay Kit, Suzhou, China). The Komas Brilliant Blue G250 staining method was used to measure the SP content (KMSP-2-W Assay Kit, Suzhou, China). The anthrone sulphate method was used to measure the SS content (KT-2-Y Assay Kit, Suzhou, China). The thiobarbituric acid (TBA) method was used to measure MDA content (MDA-2-Y Assay Kit, Suzhou, China). The Fla content was determined as described in the method of Gorinstein et al. [81]. The Pol content was determined as described in the method of Ye et al. [82].

### 4.3. Transcriptome Sequencing and Analysis

All RNA-seq experiments of both *C. vietnamensis* cultivar treatment groups (control and 3 d and 6 d PEG-treated leaf samples of two cultivars) used three biological replicates to improve the reliability of the statistical analysis. Total RNA was isolated from leaves using an RNA Preparation Pure Plant Kit (Tiangen, China) according to the manufacturer’s instructions. The cDNA library construction and transcriptome sequencing of *C. vietnamensis* leaves were completed by OE Biotech Co., Ltd. (Shanghai, China). Raw RNA-seq data were submitted to the NCBI Biological Program under SRA registration number PRJNA856766. Briefly, the mRNA was enriched using oligo(dT) magnetic beads and decomposed by the addition of fragment buffer. A single strand of cDNA was synthesized from these short fragments using random hexamer primers, followed by a second strand of cDNA using buffer, dNTPs, DNA polymerase I and RNase H. Then, the ligation products were screened by agarose gel electrophoresis, and the enriched fragments were amplified by PCR. The constructed libraries were sequenced using the Illumina HiSeq X Ten Platform (Illumina Inc., San Diego, CA, USA) [83].

Trimmomatic [84] software was used to filter out low-quality bases and N bases from the original data to obtain high-quality clean reads. Then, the clean reads obtained were compared to the reference genome (PRJNA732216) of *C. vietnamensis* using HISAT2 software [85,86]. The FPKM [87] of each gene was calculated using Cufflinks [88]. Htseqcount [89] was used to obtain the number of reads of genes in each sample, and the estimateSizeFactors function of DESeq R Package [90] was used to standardize the data. Then, DEGs with *p*-values less than 0.05 and multiple differences greater than 2 were selected, and GO- and KEGG-enrichment analyses of DEGs were performed [91].

### 4.4. Validation of DEG Expression by qRT-PCR

To verify the reliability of the RNA-seq results, a total of 18 candidate genes related to lignin biosynthesis were screened for qRT-PCR. To express data normalization processing, with the actin gene (glyceraldehyde-3-phosphate dehydrogenase, GAPDH) as the internal reference [7], the qRT-PCR primers used for DEG analysis were synthesized using Primer Premier 5.0 (Premier Biosoft International, Palo Alto, CA, USA) (Table S9) [92]. qRT-PCR was performed on a LightCycler^®^96 system (Roche Switzerland). Total RNA was extracted and purified using the above methods. ChamQTM Universal SYBR qPCR Master Mix (Novozan Biotechnology Co., Ltd., Nanjing, China) was used for PCR amplification. The PCR system was as follows: 1 µL of cDNA template, 0.5 µL each of upstream and downstream primers (10 μMol/L), 5 µL of 2 × SYBR qPCR Mix and sufficient ddH_2_O to reach a final volume of 10 μL. The PCR procedure was as follows: predenaturation at 95 °C for 30 s; 15 s at 95 °C, 30 s at 58 °C, 60 s at 72 °C, 40 cycles; 5 s at 65 °C; denatured DNA products at 95 °C. During the experiment, each gene was set up for 3 technical repetitions. The relative expression level of DEG was 2 ^−ΔΔCT^ method [93].

### 4.5. Statistical Analysis

Excel 2016 was used for data analysis and collation. IBM SPSS Statistics 20.0 software (SPSS Institute Ltd., Armonk, NY, USA) was used to analyze the variance of physiological indexes. Duncan’s multiple comparison test was used to determine the difference between means (*p <* 0.05). GraphPad Prism 5 software was used to produce the charts. TBtools software was used to create the heatmap.

## 5. Conclusions

**Figure 11:**
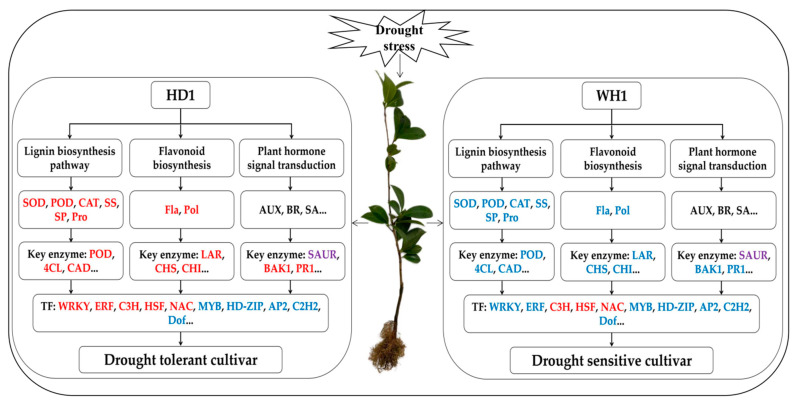
Schematic diagram of the molecular model of the main methods of drought resistance in *C. vietnamensis* seedlings of drought-tolerant and drought-sensitive cultivars under drought stress. The model is constructed based on physiological indicators measured in this paper and key genes identified in major common drought-response pathways. The model diagram was obtained by comparing drought-tolerant with drought-sensitive cultivars. Red and blue letters refer to up- and downregulation in response to drought, respectively. Purple letters refer to both up- and downregulation in response to drought.

Drought stress seriously inhibited the growth of *C. vietnamensis*, increased the antioxidant enzyme activity and MDA content of leaves and reduced the RWC. Our study showed that HD1 had stronger drought-tolerance properties than WH1. Compared with WH1, HD1 had a higher RWC, antioxidant enzyme activities, osmotic regulatory substances and flavonoid content, but a lower degree of damage to cell membrane permeability under drought stress. The number of DEGs in HD1 was less than that in WH1, indicating that the drought-resistant cultivars could maintain a highly stable gene expression level and a relatively stable cell environment. These DEGs were mainly involved in phenylpropanoid biosynthesis, flavonoid biosynthesis and plant hormone signal transduction pathways. The drought-tolerant cultivars had different regulatory strategies in phenylpropanoid biosynthesis, flavonoid biosynthesis and plant hormone signal transduction (including AUX, CTK, ET, JA, SA and BR) pathways (Figure 11), which may be the key factor for drought-resistant cultivars to have higher drought tolerance. In conclusion, our study revealed the molecular mechanism of the response to drought stress of *C. vietnamensis* and provided theoretical support for scientific cultivation and drought-resistance breeding.

## Figures and Tables

**Table 1:** Membership function values of 5 *C. vietnamensis* cultivars.

Cultivars	‘Wanhai 1’	‘Wanhai 3’	‘Wanhai 4’	‘Haida 1’	‘Haida 4’
RWC	0.0000	0.2796	0.5581	1.0000	0.7181
REC	0.0000	0.5174	0.3795	1.0000	0.9255
MDA	0.2997	0.0000	0.4412	1.0000	0.5235
SOD	0.0000	1.0000	0.1059	0.4973	0.5317
POD	0.0000	0.4896	0.2886	1.0000	0.4465
CAT	0.1656	0.7576	0.9696	1.0000	0.0000
SP	0.5955	0.8538	0.8462	1.0000	0.0000
SS	0.4839	0.8015	0.0000	1.0000	0.6483
Pro	0.3798	0.0000	0.3063	1.0000	0.6258
Fla	0.0000	1.0000	0.7868	0.6880	0.5426
Pol	0.2237	0.7244	1.0000	0.7664	0.0000
Subordinate function Mean value	0.1953	0.5840	0.5166	0.9047	0.4511
Rank	5	2	3	1	4

Note: RWC, relative water content; REC, relative electric conductivity; MDA, malonaldehyde; SOD, superoxide dismutase; POD, peroxidase; CAT, catalase; SP, soluble protein; SS, soluble sugar; Pro, proline; Fla, total flavonoids; Pol, total polyphenols.

## Data Availability

The datasets in this study were uploaded to NCBI, with the accession number PRJNA856766 (https://www.ncbi.nlm.nih.gov/sra/PRJNA856766; accessed on 7 July 2022).

## Supplementary Materials

The following supporting information can be downloaded at: https://www.mdpi.com/article/10.3390/ijms231911801/s1.
