# Supplementary material for: Physiological and Transcriptional Analysis Reveals the Response Mechanism of Camellia vietnamensis Huang to Drought Stress
Source: Int J Mol Sci. 2022 Oct 5;23(19):11801. doi: 10.3390/ijms231911801 (PMC9569630; doi:10.3390/ijms231911801)
Supplement: Supplementary file 1 [file ijms-23-11801-s001.zip › Supplementary figures.pdf]

Type of the Paper (Article)

Combined Physiological and Transcriptional Analysis Revealed the Response Mechanism of *Camellia vietnamensis* Huang to Drought Stress

Shen et al.

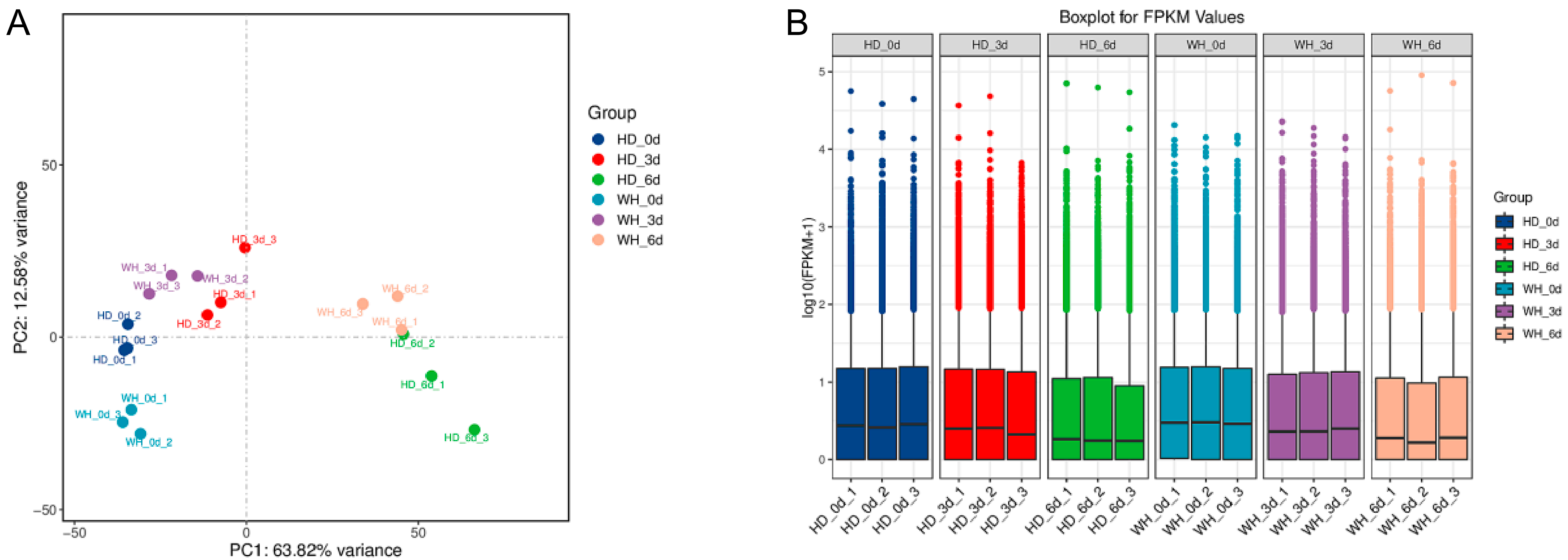

Figure S1. Principal Component Analysis (PCA) (A) and FRKM boxplot (B) of RNA sequencing of 18 HD1 and WH1 samples under drought stress.

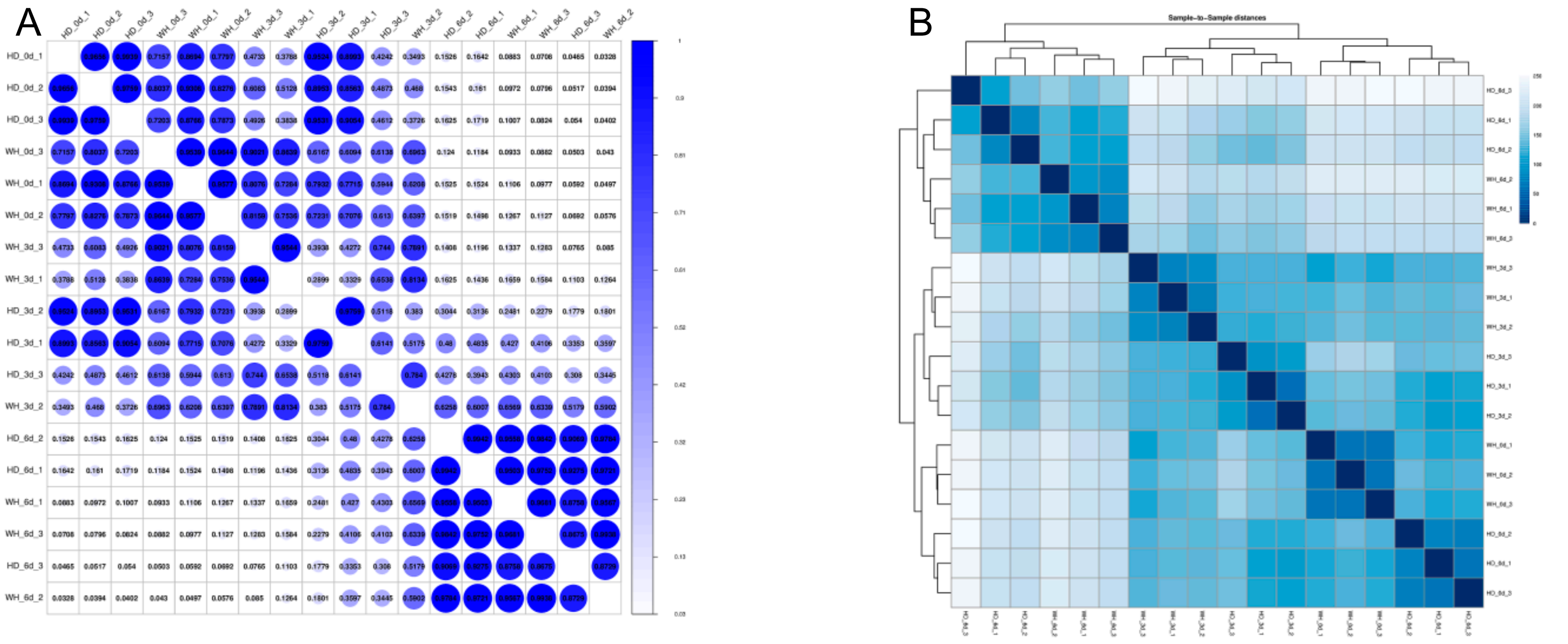

Figure S2. Correlation heat map (A) and cluster dendrogram (B) of RNA sequencing of 18 HD1 and WH1 samples under drought stress.

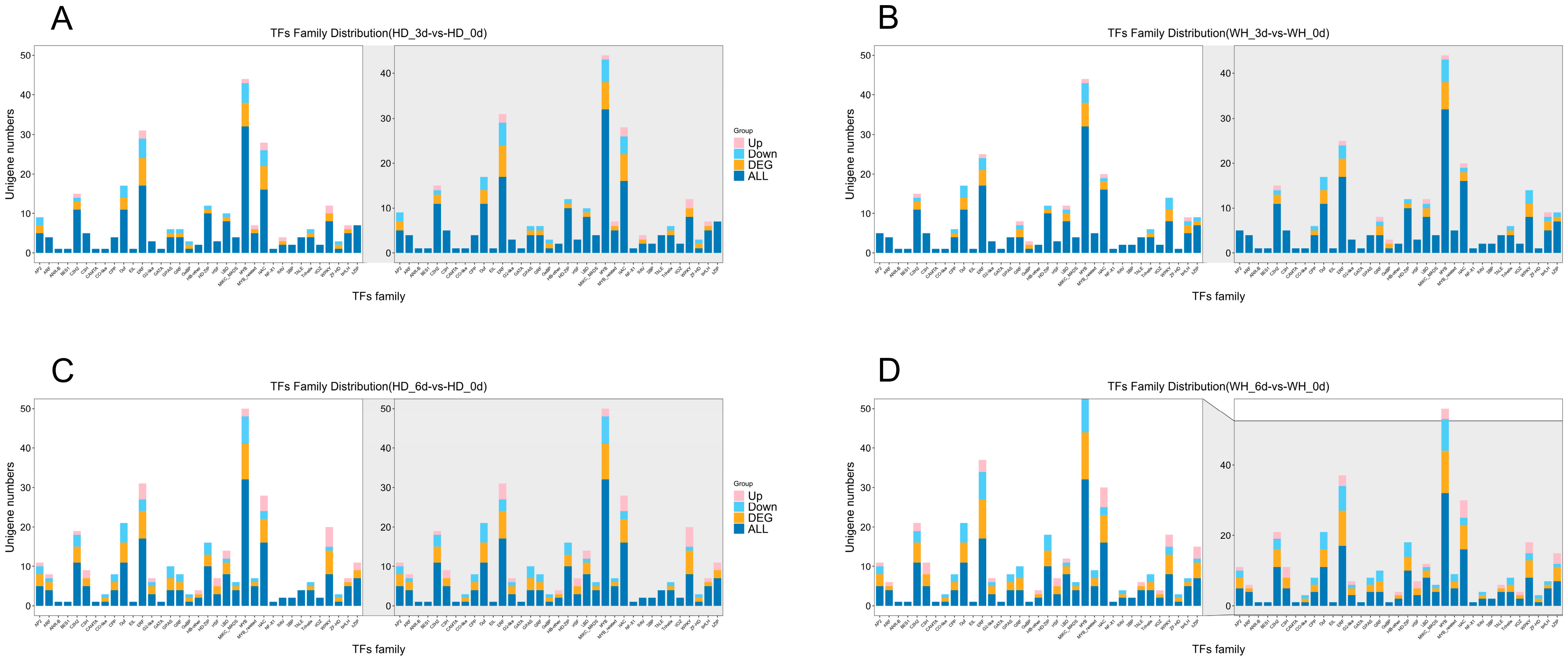

Figure S3. Transcription factor family of *C. vietnamensis* leaves under PEG stress. A. Distribution of TFs family for HD1-0d vs HD1-3d; B. Distribution of TFs family for WH1-0d vs WH1-3d; C. Distribution of TFs family for HD1-0d vs HD1-6d; D. Distribution of TFs family for WH1-0d vs WH1-6d.

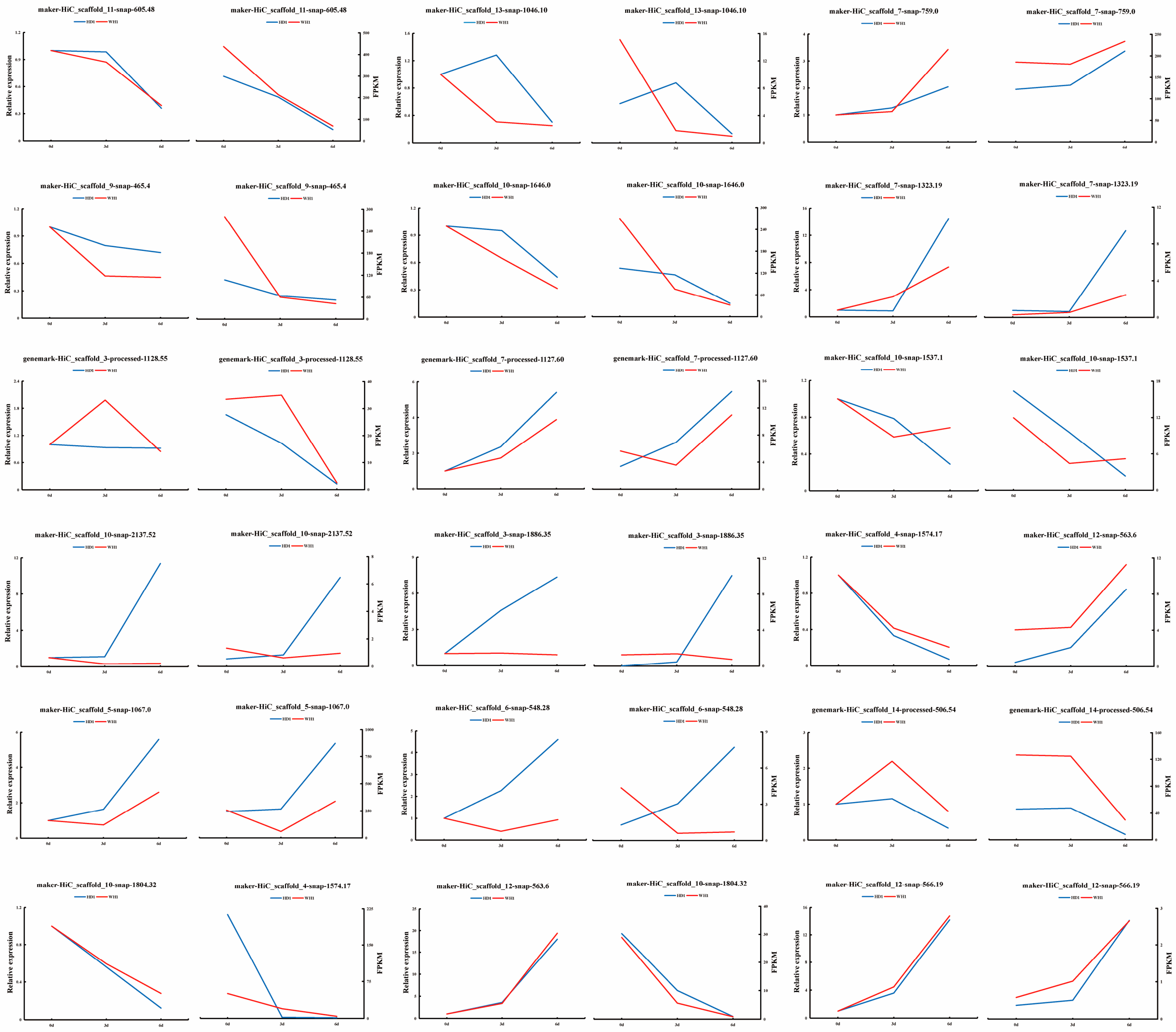

Figure S4. Expression patterns of eighteen candidate transcripts measured in HD1 and WH1 via qRT-PCR (left side) and RNA-seq (right side).
